# Corticosteroids Augment BRAF Inhibitor Vemurafenib Induced Lymphopenia and Risk of Infection

**DOI:** 10.1371/journal.pone.0124590

**Published:** 2015-04-21

**Authors:** Wiebke Sondermann, Klaus G. Griewank, Bastian Schilling, Elisabeth Livingstone, Julia C. Leyh, Natalia Rompoti, Ioana Cosgarea, Tobias Schimming, Dirk Schadendorf, Lisa Zimmer, Uwe Hillen

**Affiliations:** Department of Dermatology, University Hospital Essen, West German Cancer Center, University Duisburg-Essen and the German Cancer Consortium (DKTK), Essen, Germany; University of Queensland Diamantina Institute, AUSTRALIA

## Abstract

We have previously demonstrated an impact of the BRAF inhibitor vemurafenib on patient lymphocyte counts. In the current study, the extent to which concomitant use of corticosteroids in BRAF inhibitor treated patients affects lymphocyte counts and predisposes to infection was investigated. A cohort of 102 patients receiving either the selective BRAF inhibitor vemurafenib or dabrafenib was analyzed. The amount of patients receiving either medication with or without systemic corticosteroids (dexamethasone) was determined and lymphocyte counts before and under therapy assessed. Additionally, the number and severity of infections occurring in these groups was analyzed. Vemurafenib treatment led to a considerable decrease in lymphocyte cell counts, with 62.3% of patients having lymphopenia. Dabrafenib treated patients only rarely demonstrated lymphopenia (12.5%). Dexamethasone co-administration further diminished lymphocyte counts. Lymphopenias were observed in 84.6% of patients receiving vemurafenib and dexamethasone. In our cohort, infections were noted in 9 patients, 4 of these were severe and 2 eventually fatal. All 9 cases with infections demonstrated lymphopenia, 8 of these had received dexamethasone and 7 of these a therapy with vemurafenib. Our findings demonstrate a significant lymphopenia in patients treated with the BRAF inhibitor vemurafenib, which is further augmented by dexamethasone and predisposes to infection. If validated in other studies, risk of infection should be considered when applying corticosteroids in combination with BRAF inhibitors, in particular vemurafenib.

## Introduction

Melanoma is a particularly malignant cutaneous neoplasm and responsible for a considerable mortality worldwide[1]. In the United States, 9710 people were expected to die of melanoma in 2014[2]. Until recently, effective therapies for metastatic disease were not available. With the introduction of BRAF V600 inhibitors (BRAFi) and immunotherapies targeting CTLA-4 and PD-1, a convincing survival benefit of patients with metastasized disease receiving these treatments has been observed[3–9].

*BRAF* mutations occur in around 40–60% of melanomas and the majority of these mutations affect the V600 amino acid residue, leading to a constitutively active BRAF kinase[10]. BRAFi such as vemurafenib (VEM) and dabrafenib (DAB) have shown convincing therapeutic responses in a large number of patients receiving therapy. Although around 80% of treated patients benefit from BRAFi monotherapy[3, 11], the average duration of response before tumors become therapy resistant is around 6 months.

In patients receiving immunotherapies such as CTLA-4 or PD-1 antibodies, the response rates of patients profiting from treatment is lower than for those receiving BRAFi[7, 8, 12]. However, a proportion of patients receiving immunotherapies and showing objective responses can benefit for a number of years from therapy[13, 14]. This differs from patients receiving BRAFi monotherapy where long term responses are an exception. However, the recent introduction of regimens combining BRAF with MEK inhibitors have shown a considerably increased duration of therapy responses[15, 16].

One potential treatment strategy would be to combine immunotherapeutic agents with BRAFi. Reports of BRAFi increasing expression of tumor antigens[17] provide further support that a beneficial synergistic effect could be achieved by combination therapy. Unfortunately, one trial aiming to combine these therapies was terminated due to the high level of liver toxicities observed[18].

Recently, we described lymphopenias in patients treated with VEM[19]. This was not observed in DAB treated patients. A mean decrease in lymphocyte numbers of ~25% was noted in patients receiving VEM. The CD4 T cell population was particularly affected, showing a significant decrease in cell number. An altered phenotype with a higher proportion of CCR7+CD45RA+ (naïve) and lower proportion of CCR7+CC45RA- (central memory) cells, as well as altered cytokine profiles with lower secretion of interleukin-9 and interferon-gamma, was also noted.

Lymphocyte counts can also be affected by systemic application of corticosteroids. Corticosteroids are frequently given to oncology patients with symptomatic hepatic or brain metastases as these often significantly impede the patients´ quality of life (i.e. pain, paralysis, epileptic seizures, etc.). Corticosteroids reduce tumor-associated inflammation and can significantly and rapidly improve symptoms caused by increased tissue pressure. Corticosteroids are generally potent immunosuppressive agents and patients receiving treatment have an increased risk of infection.

The aim of our study was to retrospectively analyze to which extent patients treated with the BRAFi VEM or DAB received corticosteroids, how this treatment affected lymphocyte counts and whether this had a relevant impact on clinical parameters such as infections

## Materials and Methods

### Patient Selection

102 patients, who had received a BRAFi between May, 2010 and July, 2014 in the Department of Dermatology, University Hospital Essen were retrospectively selected for study inclusion. Patients were excluded from analysis if they had been enrolled in a clinical trial where it was not known whether they had received a BRAFi as monotherapy or in combination with a MEK inhibitor. Patients who had received BRAF and MEK inhibitor combination therapy in the context of an early access program were also excluded. The study was conducted in accordance with the principles of the Declaration of Helsinki. The Institutional Review Board of the University of Duisburg-Essen approved the study (IRB protocol number 12-4961-BO). All patients included in the study provided written informed consent. Patient written consent was also granted for medical images published in the study.

### White blood counts (WBC)

WBC were performed by routine clinical lab analysis on a Sysmex XE-5000 (Sysmex, Norderstedt, Germany) automated hematology analyzer. Lymphopenia was defined as a lymphocyte count of <1/nl (nanoliter) and was termed as “mild” or “severe” based on the CTCAE (Common Terminology Criteria of Adverse Events) version 4.0 criteria (“mild” = CTCAE grade 1 (lymphocyte count <LLN [Lower limit of normal]- 0,8/nl) or 2 (<0,8/nl- 0,5/nl), “severe” = CTCAE grade 3 (<0,5/nl- 0,2/nl) or 4 (<0,2/nl). Pre-therapy lymphocyte counts were defined as the last measured lymphocyte count within 4 weeks of initiating BRAFi therapy. The lowest lymphocyte count measured within the first 12 weeks of therapy was defined as the lymphocyte nadir. Neutrophil and eosinophil counts measured at the same time points were also assessed.

### Statistical Analyses

Various parameters of the patient cohort were investigated, including age at therapy initiation and gender, number of patients receiving VEM or DAB, as well as DEX co-medication. Additionally, impact of DEX usage prior to BRAFi therapy, especially with regard to lymphocyte counts prior to BRAFi treatment, was explored. Lymphocyte counts before and during therapy were taken into account and the amount of mild or severe lymphopenia in the following groups (all, BRAFi mono, BRAFi+DEX, VEM, VEM+DEX, DAB, DAB+DEX) documented. The difference (delta) between lymphocyte counts before and during therapy was calculated. Neutrophil and eosinophil counts were analyzed similarly to lymphocyte counts,

Chi-square and Fisher´s exact test were used to test for differences between groups for categorical variables, Mann-Whitney-U-test and one-sample t-test for continuous variables. All statistical tests were two-sided with a rejection of the null hypothesis at p<0.05. Analyses were performed with SPSS Statistics software (version 22; SPSS Chicago, IL, USA).

## Results

### Tumors and patients

In total, 102 patients with BRAFi therapy were included in the study, of which 41 (40.8%) were female and 61 (59.8%) were male. The average age at onset of therapy was 51.5 years (median: 53.2, range: 20–90). 25 (24.5%) patients received co-medication with DEX (BRAFi+DEX). VEM was taken by 66 (64.7%) patients, DEX co-administered in 13 (19.7%) cases. DAB was given to 36 (35.3%) patients, of which 12 (33.3%) received DEX co-medication (Table 1). Overall, DEX was administered in 25 cases, 22 (88%) to treat brain metastasis, 1 (4%) for liver capsule tension, 1 (4%) to improve general physique and 1 (4%) to treat vemurafenib induced arthralgia. All doses of DEX applied were supraphysiological. The average dosage given was 7.4 mg (median 8 mg, range 1–24 mg).

**Table 1:** Overview of basic patient characteristics sorted by groups.

			all patients	all BRAFi		VEM		DAB	
				mono	+ DEX	mono	+ DEX	mono	+ DEX
total			102	77	25	53	13	24	12
age at therapy initiation (years)			51,5	53.2	53.5	56.2	56,1	46.4	50.7
sex female/male	absolute		41/61	32/45	9/16	22/31	7/6	10/14	2/10
	*%*		*40*.*8/ 59*.*8*	*41*.*6/ 58*.*4*	*36/ 64*	*41*.*5/ 58*.*5*	*53*.*8/ 46*.*2*	*41*.*7/ 58*.*3*	*16*.*7/ 83*.*3*
no LP	absolute		51	41	10	20	2	21	8
	*%*		*50*	*53*.*2*	*40*	*37*.*7*	*15*.*4*	*87*.*5*	*66*.*7*
LP	absolute		51	36	15	33	11	3	4
	*%*		*50*	*46*.*8*	*60*	*62*.*3*	*84*.*6*	*12*.*5*	*33*.*3*
LP grade 1+2	absolute		37	30	7	28	5	2	2
	*%*		*72*.*5*	*83*.*3*	*46*.*7*	*84*.*8*	*45*.*5*	*66*.*7*	*50*
LP grade 3+4	absolute		14	6	8	5	6	1	2
	*%*		*27*.*5*	*16*.*7*	*53*.*3*	*15*.*2*	*54*.*5*	*33*.*3*	*50*
mean lymph. prether. /nl			1.37	1.45	1.09	1.37	1.05	1.64	1.14
mean lymph. under ther. /nl			1.11	1.21	0.82	1.02	0.59	1.62	1.08
mean delta lymphocytes/nl			-0.25	-0.25	-0.27	-0.35	-0.46	-0.2	-0.06
absolute number of infections			9	2	7	1	6	1	1

LP = lymphopenia; grades 1–4 according to CTCAE criteria version 4.1; BRAFi = BRAF inhibitor; VEM = vemurafenib; DAB = dabrafenib; DEX = dexamethasone; delta lymphocytes = difference between pretherapeutic lymphocyte count and lymphocyte count under therapy.

### Lymphopenia in the BRAFi group

Pre-therapy lymphocyte counts were significantly lower in the BRAFi+DEX group, than in the BRAFi group (1.09/nl vs. 1.45/nl, p = 0.006). The average difference in lymphocyte counts before and in the first 12 weeks of therapy did not differ significantly between the BRAFi and BRAFi+DEX groups (p = 0.06), however the nadir in the BRAFi+DEX group was significantly lower than in the BRAFi group (0.82/nl versus 1.21/nl, p = 0.012)-

Overall, 51 (50%) of patients developed a lymphopenia. Of these, 36 (46.8% of 77 patients) were in the BRAFi group and 15 (60% of 25 patients) in the BRAFi+DEX group. A severe lymphopenia (CTCAE grade 3 or 4) occurred in 14 of 51 (27.5%) patients. Severe lymphopenias were identified significantly more often in the BRAFi+DEX group (8 of 15 [53.3%]) than in the solely BRAFi treated group (6 of 36 [16.7%], p = 0.02, Table 1).

In 21 of 25 BRAFi+DEX patients (84%) DEX treatment had started before initiating BRAFi therapy and of these 15 (71.4%) already demonstrated lymphopenia prior to BRAFi therapy.

### Lymphopenia in VEM and DEX subgroups

**Fig 1:**
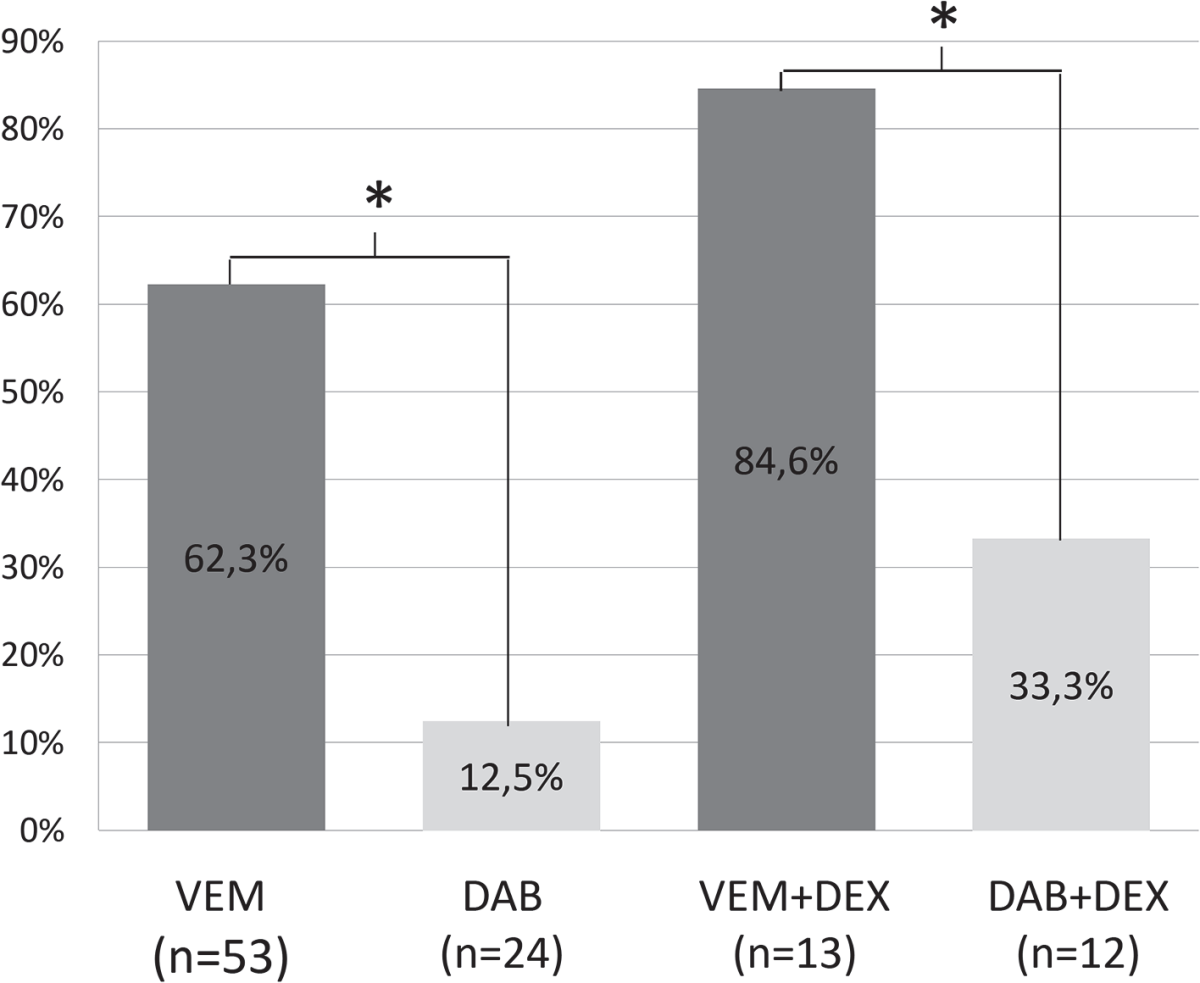
Frequency of lymphopenias in various treatment groups. Shown are the frequencies of lymphopenias patients treated receiving vemurafenib (VEM) or dabrafenib (DAB), with or without concomitant corticosteroid therapy (dexamethasone—DEX). * = p < 0.05 marking statistically significant differences between groups; n = number of patients

**Fig 2:**
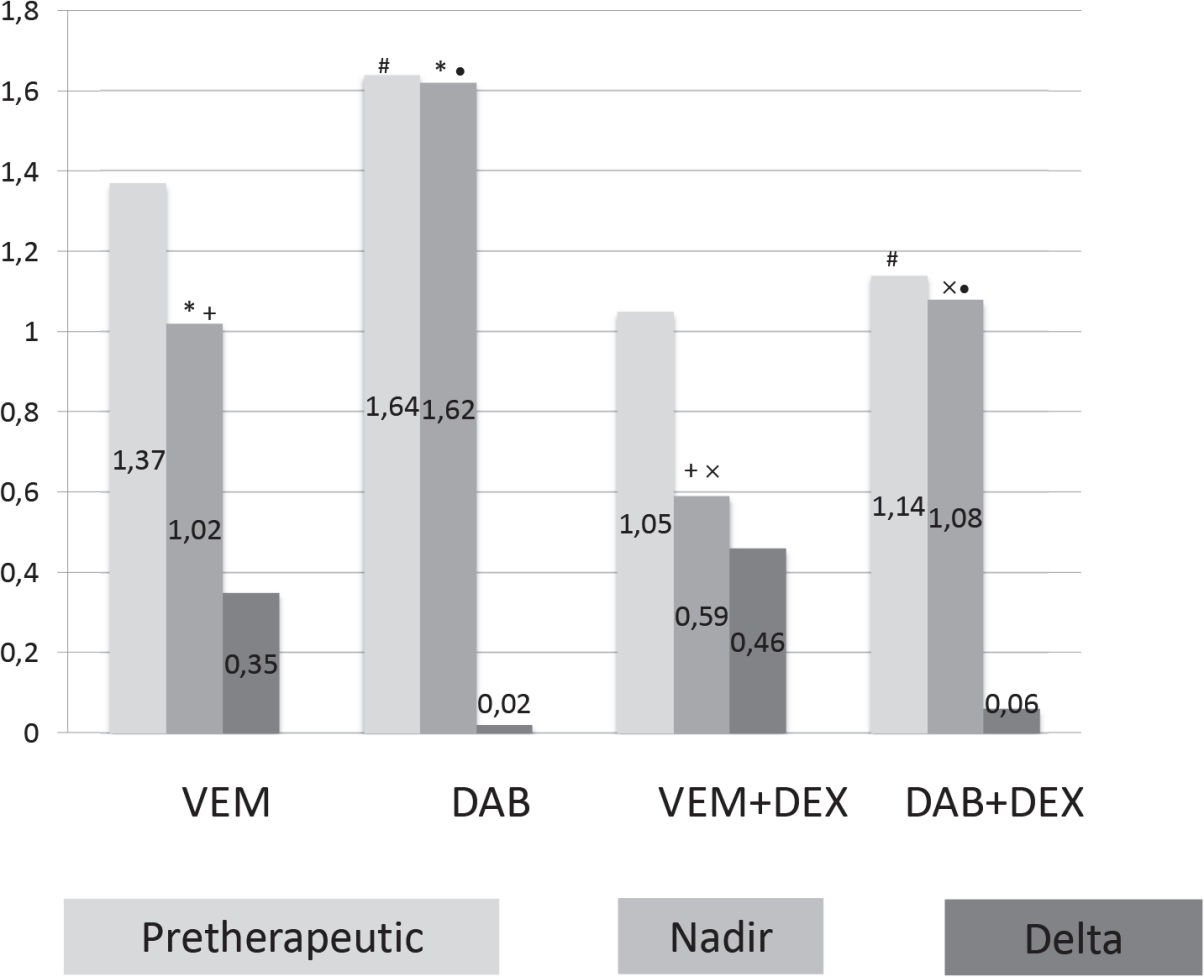
Mean lymphocyte counts before and during therapy. Shown are the mean overall lymphocyte counts before and during therapy according to treatment group. VEM- vemurafenib; DAB- dabrafenib; DEX- dexamethasone; delta lymphocytes = difference between pretherapeutic lymphocyte count and lymphocyte count under therapy; corresponding symbols (*, #, +, x, °) above bars indicate statistical significant difference between groups (p < 0.05).

Comparisons between different groups (VEM, VEM+DEX, DAB, DAB+DEX—Figs 1 and 2), only showed a significant difference (p = 0.045) in pre-therapeutic lymphocyte values between the DAB+DEX group (1.14/nl) and the DAB group (1.64/nl), but significant differences in lymphocytes counts were found between VEM and DAB groups while under BRAFi therapy (VEM vs. DAB, 1.02/nl vs. 1.62/nl [p<0.01]; VEM+DEX vs. DAB+DEX, 0.59/nl vs. 1.08/nl, [p = 0.022]). Comparisons of lymphocyte counts with and without DEX treatment were also significant (VEM vs. VEM+DEX, 1.02/nl vs. 0.59/nl [p = 0.07] and DAB vs. DAB+DEX, 1.62/nl vs. 1.08/nl [p = 0.038]).

Comparisons in terms of changes in lymphocyte numbers during the course of therapy showed no significant differences between groups (Fig 2).

**Fig 3:**
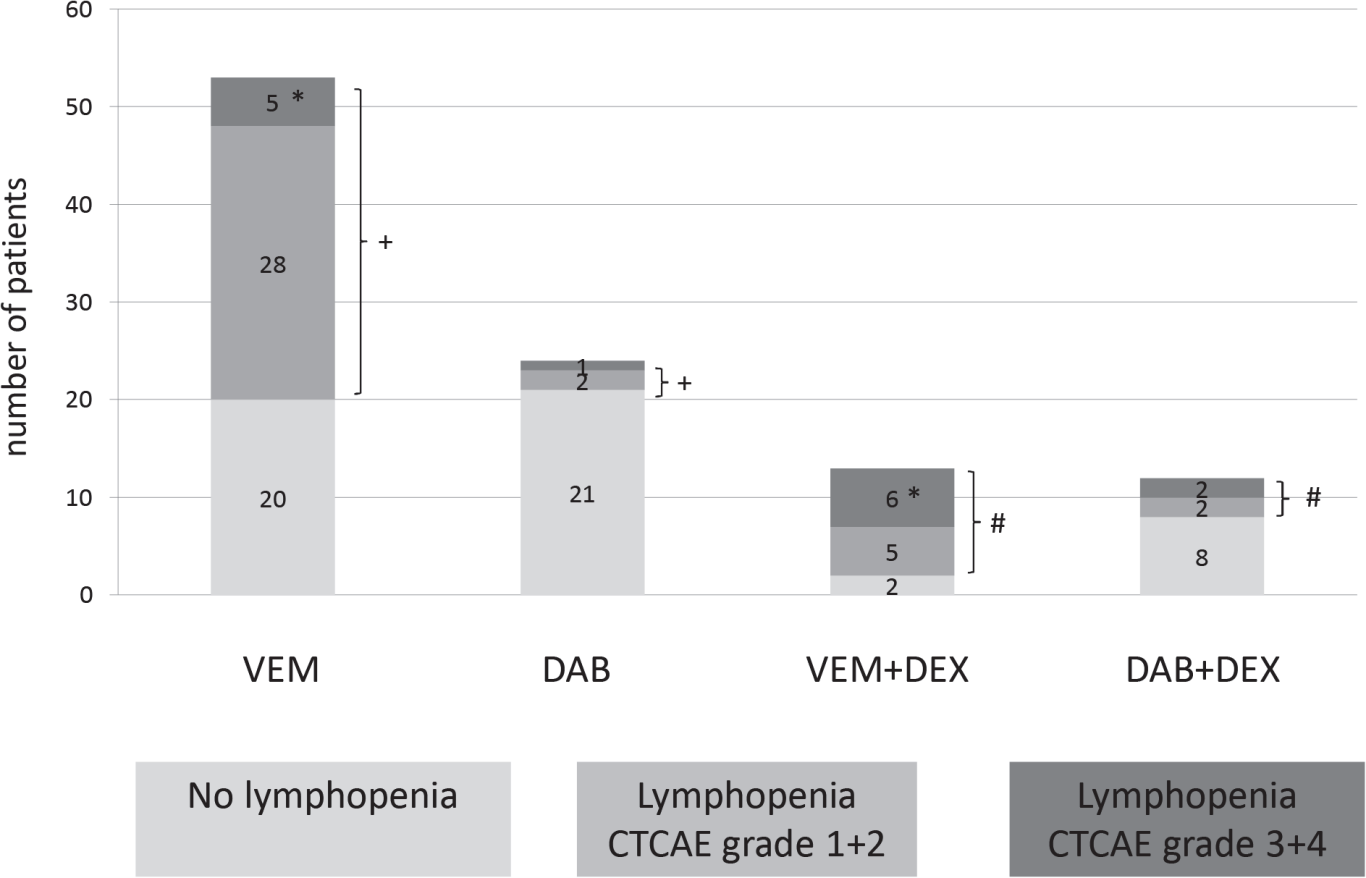
Distribution and degree of lymphopenia in treatment groups. Shown are the frequency and grade of lymphopenias in patients receiving BRAF inhibitor (vemurafenib or dabrafenib) monotherapy or those receiving both BRAF inhibitor and dexamethasone. VEM- vemurafenib; DAB- dabrafenib; DEX- dexamethasone; delta lymphocytes- difference between pretherapeutic lymphocyte count and lymphocyte count under therapy; CTCAE = Common Terminology Criteria of Adverse Events, version 4.0; corresponding symbols indicate statistically significant difference between groups (p < 0.05).

The frequency of lymphopenias was significantly higher in VEM than DAB and VEM+DEX than DAB+DEX treated patients (p<0.0001 and p = 0.009, respectively). Significant differences were not observed between VEM and VEM+DEX or DAB and DAB+DEX groups (Figs 1 and 3).

Severe lymphopenias (CTCAE grade 3 or 4) were detected significantly more often in the VEM+DEX group than in the VEM group (p = 0.01). Comparisons between other groups found no significant differences (Fig 3).

### Infections under therapy

**Fig 4:**
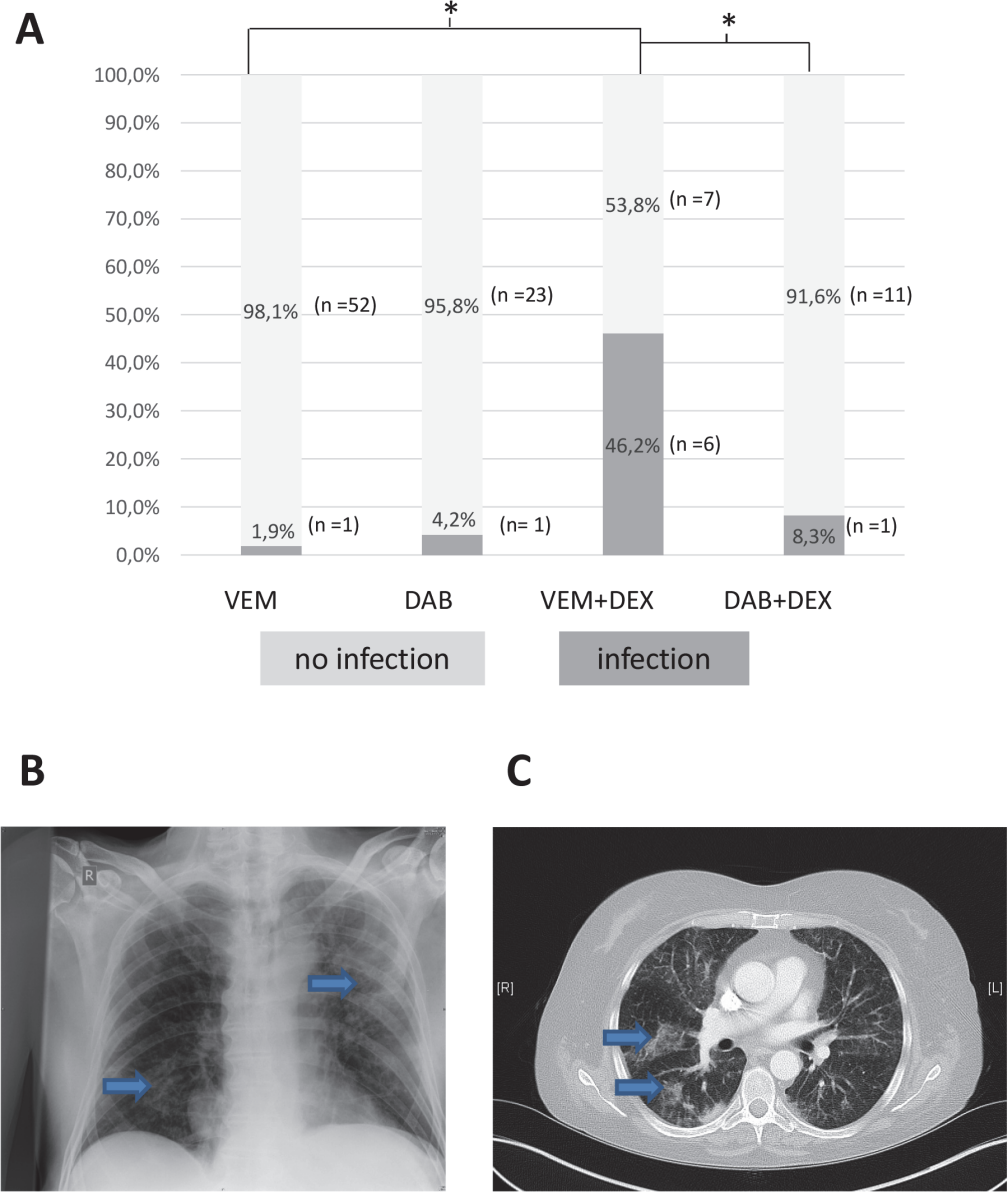
Images of infections occurring under BRAFi and DEX therapy. (A) The absolute number and percentage of patients developing infections in different treatment groups is shown. (B) Chest x-ray of a patient under therapy with vemurafenib and dexamethasone showing reticular pulmonary infiltrates (blue arrows). (C) CT scan of the thorax of a patient under therapy with vemurafenib and dexamethasone showing atypical pulmonary infiltrates (blue arrows). VEM = vemurafenib; DAB = dabrafenib; DEX = dexamethasone; * = p< 0.05 marking statistically significant difference between groups; n = number of patients.

Nine patients experienced an infection while under BRAFi therapy. Two of these were severe pneumonia resulting in patient death (Fig 4). An additional patient with pneumonia had a concurrent severe gastrointestinal infection. One patient developed CTCAE grade 3 pyrexia with procalcitonin (PCT) levels increasing massively without an identifiable source of infection. Five mild infections were noted consisting of either cellulitis or mucosal candida infection. Both patients dying from pneumonia had limited lung metastasis. Lymphopenia was observed in all nine cases. Eight of these (89%) had received DEX and seven of these (88%) a therapy with VEM (Table 2).

**Table 2:** Overview of patients’ characteristics with infections under therapy.

patient number	1	2	3	4	5	6	7	8	9
age at therapy onset	61	27	48	77	79	78	40	41	62
sex	male	female	female	male	male	male	male	female	male
Infection type	cellulitis	oropharyngeal candidiasis	pneumonia	pneumonia	oropharyngeal candidiasis	pneumonia and salmonellosis	oropharyngeal candidiasis	urinary tract infection	sepsis with unknown focus
Infection severity	benign	benign	severe	severe	benign	severe	benign	benign	severe
BRAFi	VEM	VEM	VEM	VEM	VEM	VEM	VEM	DAB	DAB
previous therapies	none	none	1 dose of Ipilimumab	none	none	none	none	2 different chemo-Tx	none
DEX dose (mg)	0	6	8	6	4	8	24	0	2
DEX prior to BRAFi	-	no	yes	yes	yes	yes	yes	-	yes
LP prior to BRAFi initiation under DEX	-	no	yes	yes	yes	yes	yes	-	yes
prether. lymph./nl	1.13	1.74	0.6	0.5	1.07	1.49	0.1	0.31	0.81
nadir lymph./nl	0.96	1.61	0.2	0.09	0.45	0.5	0.18	0.43	0.18
delta lymph./nl	-0.17	-0.13	-0.4	-0.41	-0.62	-0.99	0.08	0.12	-0.63
CTCAE grade LP	1	0	3	4	3	2	4	3	4
Infection outcome	resolved	resolved	fatal	fatal	resolved	resolved	resolved	resolved	resolved
Overall outcome	dead	alive	dead	dead	dead	dead	dead	dead	dead

LP = lymphopenia; grade 1–4 according to CTCAE criteria version 4.1; BRAFi = BRAF-inhibitor; VEM = vemurafenib; DAB = dabrafenib; DEX = dexamethasone; delta lymphocytes = difference between pretherapeutic lymphocyte count and lymphocyte count under therapy; severe infection = life-threatening infection.

In the VEM+DEX group 6 out of 13 patients (46.2%) developed an infection. This rate was significantly higher than in both the VEM group (1 out of 53 patients [1.9%], p<0.0001) and the DAB+DEX group (1 out of 12 [8.3%], p = 0.035), as shown in Fig 4A. Comparisons of groups receiving either VEM or DAB as monotherapy showed no significant difference in infection rate (p = 0.56), nor did a comparison of DAB mono with DAB+DEX (p = 0.61). The dose of DEX given to patients with an infection was not significantly higher than those without an infection (8.29 mg to 7.11 mg, respectively, p = 1.0). Table 3 shows the lymphocyte counts before and under therapy as well as the mean difference in lymphocyte counts in the group with infections and without.

**Table 3:** Mean lymphocyte counts in patient groups with infections.

	Lymphocytes		
	mean/nl		
Infection	pretherapy	nadir	delta
no (n = 93)	1.41	1.17	-0.24
yes (n = 9)	0.86	0.51	-0.35
**patients with infection**			
VEM (n = 1)	1.13	0.96	-0.17
VEM+DEX (n = 6)	0.92	0.51	-0.41
DAB (n = 1)	0.31	0.43	0.12
DAB+DEX (n = 1)	0.81	0.18	-0.63
**severe infection**	0.85	0.24	-0.61
**mild infection**	0.87	0.73	-0.14

VEM = vemurafenib; DAB = dabrafenib; DEX = dexamethasone; delta lymphocytes = difference between pretherapy lymphocyte count and lymphocyte count under therapy; severe infection = life-threatening infection.

To assess the effect of lymphopenia duration on infection, patients were grouped into those known as being lymphopenic for ≥ or < 4 weeks. Lymphopenia ≥ 4 weeks was more frequent in patients with infections (7/8 = 87.5%) compared to patients where no infections were observed (28/43 = 65.1%). The difference however, was not statistically significant.

### Neutrophils and eosinophils in treatment groups

An individual analysis of neutrophil and eosinophil counts in different groups showed that DEX therapy led to statistically significant increases in neutrophil and decreases in eosinophil counts (p<0.0001, S1 Table and S1 Fig). Treatment with VEM or DAB caused a mild, statistically not significant decrease in neutrophil counts and had minimal effects on eosinophil counts (S1 Table and S1 Fig).

Selective analysis of patients receiving DEX co-medication with or without infections showed no statistically significant difference in neutrophil counts before (9.64/nl vs. 10.01/nl, respectively) or under BRAFi therapy (7.56/nl vs. 4.68/nl, respectively). Comparable analysis of eosinophil counts in DEX treated patients with or without infections showed no statistically significant difference before BRAFi therapy (0.02/nl vs. 0.05/nl, respectively), however the difference in eosinophil nadir under BRAFi treatment was statistically significant (0.02/nl vs. 0.06/nl, respectively, p = 0.041).

### Previous therapies

Overall 77 patients (75.5%) had not previously received systemic therapies for metastasized melanoma prior to BRAFi therapy. Of the 25 patients with prior therapy, 18 (72%) had received conventional chemotherapeutic agents, 7 patients (28%) targeted therapies (non-BRAFi) and 6 patients (24%) immunotherapeutic agents. No statistically significant difference in the median number of previous therapies was observed between patients with and without infection.

## Discussion

In our study, we recognize a considerable amount of patients who experienced severe lymphopenias while under BRAFi treatment. Fitting our initial study[19], lymphopenias were primarily noted in VEM treated patients and only rarely affected patients receiving DAB (62.3 vs. 12.5%). The percentage of lymphopenias was further increased in both groups if patients additionally received systemic corticosteroid therapy (84.6% and 33.3%, respectively). This makes it evident, that in patients receiving either corticosteroids, VEM or in particular the combination of VEM and corticosteroids, lymphopenias frequently occur.

Lymphopenia, especially severe lymphopenia, is a potentially dangerous condition. It impedes the immune system in establishing a protective immune response, thus predisposing patients to infections with a variety of different pathogens. It is well known that systemic administration of corticosteroids can lead to immunosuppression, by causing lymphopenia and other mechanisms[20, 21]. However, patients with clinical symptoms of metastasis due to increased tissue pressure can benefit strongly from systemic corticosteroids. Corticosteroids are a potent treatment for pain, nerve paralysis or epileptic seizures due to tumor induced pressure in the central nervous system. Another indication for therapy with corticosteroids in oncology patients is severe pain caused by metastasis related liver capsule tension[22]. In these situations, the moderate increase in risk of infection is a minor concern compared to the considerable and rapid treatment benefit that can be achieved.

Identifying signs of infection can be difficult in patients with advanced disease. As classical clinical signs such as reduced general physique, fever, leucocytosis and elevated CRP (C-reactive protein) levels frequently occur in tumor patients without infection, due to a high tumor burden[23], identification of a pathogen-induced infection may be delayed and only noted at a relatively late stage. This combined with a general state of immunosuppression caused by advanced tumor burden can lead to a particularly severe and potentially fatal course of infection. Patients who develop lymphopenia while receiving VEM and systemic corticosteroids could be at a particularly increased risk for complications.

Understanding the extent to which BRAFi and corticosteroid therapy affect lymphocyte counts would be valuable, in particular if it is a simple additive effect, or if the combination results in a more drastic decrease in lymphocyte cell numbers. Given that the patients did not have a baseline lymphocyte count prior to the initiation of dexamethasone the effect of dexamethasone on lymphocyte counts could not be directly evaluated. It is however apparent, that these patients had lower lymphocyte counts at BRAFi therapy initiation (Fig 2), which argues for an effect of DEX. The additional decrease in lymphocyte counts after BRAFi treatment initiation (0.27/nl) is comparable to the decrease in the solely BRAFi treated group (0.25/nl), arguing that the effects of BRAFi and DEX observed are primarily additive.

The potential effect of neutrophil and eosinophil counts on infections was also addressed in our study. Clear neutrophil count increases and eosinophil count decreases under DEX therapy were noted. VEM and DAB treatment decreased neutrophil cell counts mildly and exerted only minor effects on eosinophil cell counts. Overall, the effect of VEM and DAB on eosinophil and neutrophil counts was relatively comparable. DEX treated patients with and without infection had comparable neutrophil counts within the normal range before and under BRAFi therapy, excluding low neutrophil counts as a causative factor for infection in our study.

A modest statistical significance for lower eosinophil counts in BRAFi and DEX-treated patients with infections than similarly treated patients without infections was noted. This could imply a contribution of low eosinophil counts to infections. However, as VEM or DAB treatment generally showed comparable, minimal effects on eosinophil counts, it seems unlikely effects on eosinophils are responsible for the strong infection bias observed in the VEM and DEX treated patient group. Larger studies analysing more patients will be required to assess if eosinophil counts are truly a relevant factor in these settings.

An interesting aspect that merits consideration is the effect the induced lymphopenias might have on therapy designs where BRAFi are combined with immunotherapies such as CTLA-4 and PD-1 antibodies. In clinical studies of such treatment regimens, it would seem appropriate to routinely analyse patient lymphocyte counts and in cases where lymphopenia is noted, further application of the immunotherapeutic agent or continuation of systemic steroid therapy should be considered carefully. As seen in the current and our previous study[19], the effects of different BRAFi may vary strongly, indicating that effects observed for one substance, such as VEM, should not be seen as representative for other agents of the same group, e.g. DAB. Based on the results of this and our previous study [19], it would seem apparent, that combining DAB with immunotherapies would have greater potential, as lymphocyte counts and function are seemingly not, or just mildly, affected in comparison to VEM. Not compromising the potential to initiate a strong lymphocytic response would appear vital if patients should profit from combined BRAF inhibitor and immunostimulative therapies.

Even though the observed infection rate was still relatively low (9%), four of these infections were severe and two even fatal. Both fatal infections occurred in patients receiving VEM+DEX with extremely low lymphocyte nadirs (0.2/nl and 0.09/nl). Due to the small number of patients, it cannot be excluded that the occurrence of lymphopenia and severe infections in the same treatment group is purely coincidental. DEX alone is usually given to more severely ill oncology patients. This could be associated with an increased infection rate independent of BRAF inhibitor treatment or lymphopenia. However, the impressive overall frequency of patients with VEM+DEX treatment that developed infections (46.2%, 6 of 13) compared to all other groups, including DAB+DEX, where infections were the exception (Fig 4A), does strongly support that the combination of VEM and DEX increases the risk of infection. At least in our cohort, these infections occasionally proved to be fatal and prophylactic measures need to be implemented early in the future.

This study is not without limitations. Due to the limited number of patients included, only a few could be analysed that had severe infections. Larger case numbers would be required to allow a significant analysis of these cases. Another aspect that made the evaluation and data interpretation more difficult was that most patients (84%) receiving BRAFi+DEX had already received corticosteroids before initiating BRAFi therapy. Lymphocyte counts prior to corticosteroid therapy were generally not available. Certain infections such as oropharyngeal candidiasis could be primarily DEX induced. A valuable control group would consist of patients having received only DEX, however for obvious ethical reasons such data was not available for a metastasized melanoma patient cohort. To be able to further evaluate the combined effect of BRAFi and corticosteroids on lymphocyte counts and validate our current observations, larger numbers of patients would need to be analysed in an—ideally—prospective study.

Overall, we believe our data points towards an additive effect of the BRAFi VEM with DEX in inducing lymphopenias in treated patients. This effect could be clinically relevant as it might predispose patients with metastatic melanoma, who are already at increased risk for infections, even further. If validated in larger studies, this side effect of BRAFi and corticosteroid treatment should be considered when applying such treatments to advanced oncology patients. While this doesn´t necessarily mean BRAFi generally shouldn´t be combined with corticosteroids, it does suggest that lymphocyte counts should be monitored closely before and during therapy and patients be assessed for clinical signs of infections. In cases where a significant lymphopenia is observed or eminent, one should either try to avoid combining corticosteroids and BRAFi, or consider treating affected patients with a BRAFi having a less profound effect on patients´ lymphocytes.

## Supporting Information

S1 Fig Neutrophil and eosinophil counts before and during therapy. Shown are the mean overall neutrophil (A) and eosinophil (B) counts before and during therapy according to treatment group. VEM- vemurafenib; DAB- dabrafenib; DEX- dexamethasone; delta = difference between pretherapeutic count and count under therapy; corresponding symbols above bars indicate statistically significant difference between groups (p < 0.05)
(PDF)

S1 Table Mean neutrophil and eosinophil counts in patient groups with infections. (DOCX)
