# Supplementary figures and images for: Corticosteroids Augment BRAF Inhibitor Vemurafenib Induced Lymphopenia and Risk of Infection
Source: PLoS One. 2015 Apr 21;10(4):e0124590. doi: 10.1371/journal.pone.0124590 (PMC4405567; doi:10.1371/journal.pone.0124590)

# Supplemental Figure 1. Neutrophil and eosinophil counts before and during therapy

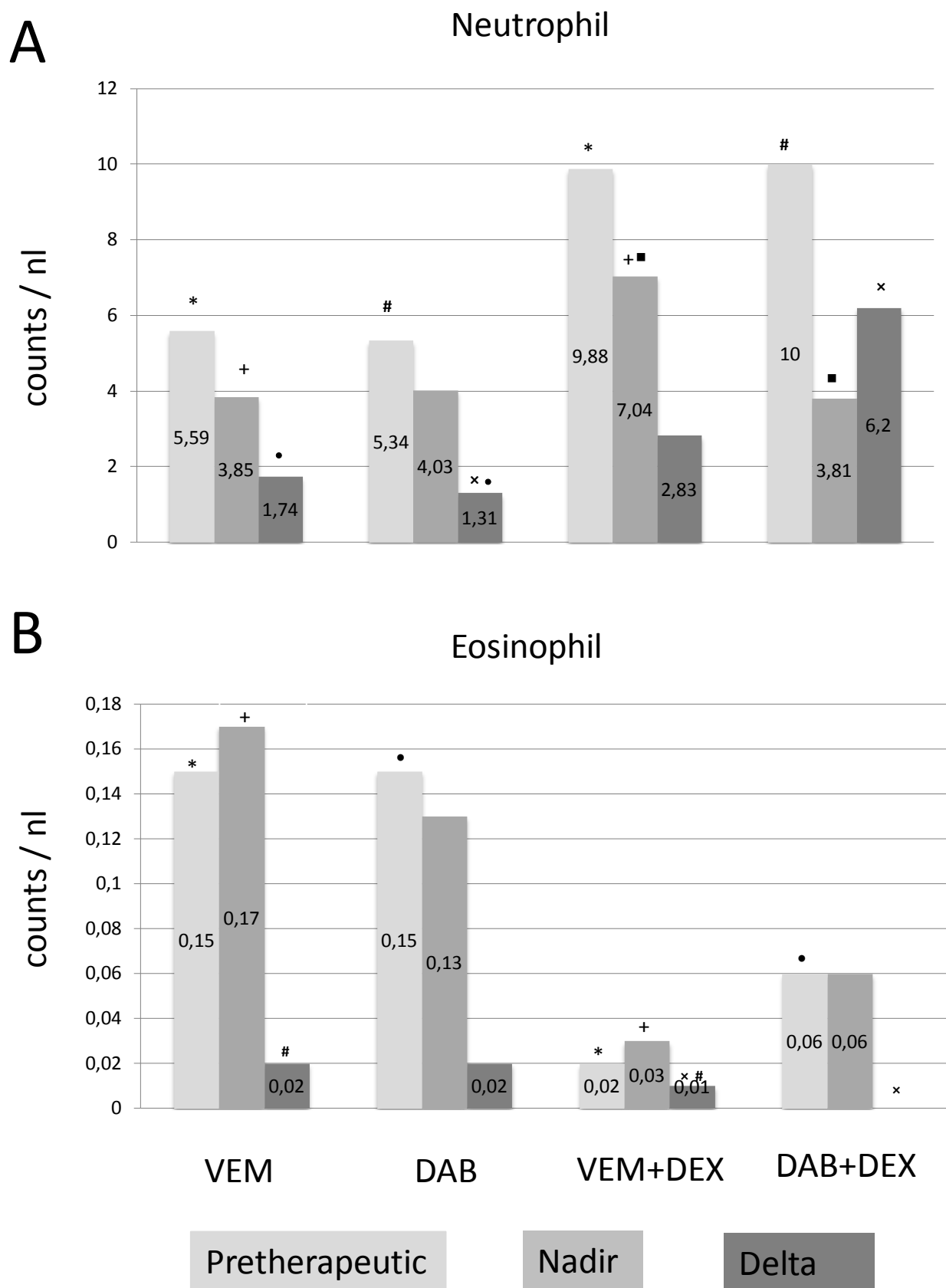

Supplement: S1 Fig — Shown are the mean overall neutrophil (A) and eosinophil (B) counts before and during therapy according to treatment group. VEM- vemurafenib; DAB- dabrafenib; DEX- dexamethasone; delta = difference between pretherapeutic count and count under therapy; corresponding symbols above bars indicate statistically significant difference between groups (p < 0.05) (PDF) [file pone.0124590.s001.pdf]
