# Supplementary material for: Corticosteroids Augment BRAF Inhibitor Vemurafenib Induced Lymphopenia and Risk of Infection
Source: PLoS One. 2015 Apr 21;10(4):e0124590. doi: 10.1371/journal.pone.0124590 (PMC4405567; doi:10.1371/journal.pone.0124590)
Supplement: S1 Table — (DOCX) [file pone.0124590.s002.docx]

**Supplemental Table 1:**

**Mean neutrophil and eosinophil counts in patient groups with infections**

|  | **Neutrophils**  **mean/nl** | | | **Eosinophils**  **mean/nl** | | |
| --- | --- | --- | --- | --- | --- | --- |
| **Infection** | **pretherapy** | **nadir** | **delta** | **pretherapy** | **nadir** | **delta** |
| no (n=93) | 6.39 | 4.04 | -2.35 | 0.13 | 0.14 | 0.01 |
| yes (n=9) | 8.67 | 6.93 | -1.74 | 0.03 | 0.01 | -0.01 |
|  |  |  |  |  |  |  |
| **patients with infection** |  |  |  |  |  |  |
| VEM (n=1) | 6.2 | 4.77 | -1.43 | 0.07 | 0 | -0.07 |
| VEM+DEX (n=6) | 9.94 | 8.3 | -1.64 | 0.03 | 0.02 | -0.01 |
| DAB (n=1) | 4.41 | 4.7 | -0.29 | 0 | 0 | 0 |
| DAB+DEX (n=1) | 7.81 | 3.13 | -4.68 | 0.01 | 0.02 | 0.01 |
| **severe infection** | 10.3 | 7.89 | -2.41 | 0.01 | 0.01 | 0 |
| **mild infection** | 7.37 | 6.17 | -1.2 | 0.04 | 0.02 | -0.02 |

VEM = vemurafenib; DAB = dabrafenib; DEX = dexamethasone; delta = difference between pretherapy neutrophil and eosinophil count and neutrophil and eosinophil count under therapy; severe infection = life-threatening infection.
